# Opposite transcriptional regulation of integrated *vs* unintegrated HIV genomes by the NF-κB pathway

**DOI:** 10.1038/srep25678

**Published:** 2016-05-11

**Authors:** Sylvain Thierry, Eloïse Thierry, Frédéric Subra, Eric Deprez, Hervé Leh, Stéphanie Bury-Moné, Olivier Delelis

**Affiliations:** 1 LBPA, ENS Cachan, CNRS UMR8113, IDA FR3242, Université Paris-Saclay, F-94235 Cachan, France

## Abstract

Integration of HIV-1 linear DNA into host chromatin is required for high levels of viral expression, and constitutes a key therapeutic target. Unintegrated viral DNA (uDNA) can support only limited transcription but may contribute to viral propagation, persistence and/or treatment escape under specific situations. The molecular mechanisms involved in the differential expression of HIV uDNA *vs* integrated genome (iDNA) remain to be elucidated. Here, we demonstrate, for the first time, that the expression of HIV uDNA is mainly supported by 1-LTR circles, and regulated in the opposite way, relatively to iDNA, following NF-κB pathway modulation. Upon treatment activating the NF-κB pathway, NF-κB p65 and AP-1 (cFos/cJun) binding to HIV LTR iDNA correlates with increased iDNA expression, while uDNA expression decreases. On the contrary, inhibition of the NF-κB pathway promotes the expression of circular uDNA, and correlates with Bcl-3 and AP-1 binding to its LTR region. Finally, this study identifies NF-κB subunits and Bcl-3 as transcription factors binding the HIV promoter differently depending on viral genome topology, and opens new insights on the potential roles of episomal genomes during the HIV-1 latency and persistence.

Integration of the HIV genome is an essential step of the retroviral cycle, supporting massive production of viral particles. Strikingly, integrated viral DNAs (iDNAs) only represent a minor part of reverse-transcribed genomes, that remains mainly under unintegrated viral forms (uDNAs) at early times post-infection as well as during untreated chronic infection^1^,^2^. Unintegrated HIV genomes mainly include linear DNAs (DNA_L_) that are quickly degraded, and circular DNAs containing one or two long terminal repeats (1-LTRc and 2-LTRc, respectively)^3^,^4^. Conversely, 1-LTRc and 2-LTRc episomal DNAs remain intrinsically stable and only diminish through cell death or division (*e.g.* following T cell activation)^4^,^5^. Several studies have demonstrated the stability of uDNAs in non-dividing primary macrophages and resting CD4 T cells^6^,^7^,^8^,^9^. This stability is supported by clinical trials highlighting the high levels and persistence of 2-LTRc in HIV-1 controllers^10^,^11^.

Until recently, uDNAs were considered as “dead-end products” of reverse transcription. However, several reports have now established that circular uDNAs can support low levels of HIV expression^7^,^8^,^12^,^13^,^14^,^15^, can constitute a reserve substrate for *de novo* integration^16^, and be a source of infectious virus^17^,^18^,^19^ (for a review^20^). Therefore, HIV uDNAs should be considered as potential reserve genomes that could be involved in persistence and treatment escape. Although expression of uDNAs can be several orders of magnitude lower than that of an integrated provirus^13^,^15^, it can lead to the expression of accessory proteins such as Nef and Tat^14^,^15^. Importantly, this low level of Nef expression is sufficient to down-regulate CD4 expression on host cell surfaces and to induce T cell activation^7^, highlighting the importance of uDNA expression on HIV-host interaction.

LTR-mediated expression from HIV iDNA is well documented. Host transcription factor mobilization and chromatin decondensation are required for robust HIV transcription, so that HIV post-integration latency is considered as a transcription factor restriction phenomenon^21^,^22^. Notably, HIV-1 LTR contains binding sites for several inducible transcription factors, including NFκB, NFAT or AP-1 (c-Jun/cFos family members) (for reviews^21^,^23^,^24^). HIV-1 transcription is thus tightly coupled to cell type and activation status. After appropriate stimulation of CD4^+^ T cells, the active form of NF-κB (p50-p65 heterodimer) translocates to the nucleus and stimulates viral expression. In these T cells, the p50-p50 homodimer is generally considered as a repressive form of NF-κB, associated with impaired viral transcription^23^. However, interaction of the NF-κB p50-p50 homodimer with the IκB-like protein, Bcl-3, can switch the balance from inhibition to activation of HIV transcription^25^,^26^,^27^. This interaction may be particularly important to modulate HIV expression in differentiated macrophages that express a constitutive nuclear pool of NF-κB, mainly constituted of p50^28^. This illustrates the importance of cell-specific factors as well as protein partner interactions on the regulation of HIV expression.

Interestingly, NF-κB and AP-1 pathways have been recently identified as pathways targeted by two distinct restriction factors, TRIM5α and BST2/tetherin, through the activation of the growth factor-β-activated kinase 1 (TAK-1)^29^,^30^,^31^,^32^. This activation by TRIM5α and BST2/tetherin thus appeared as part of the host defense signaling. However, viral components, gp41^33^ and Vpr^34^, also activate the same upstream signaling partner, TAK-1, to stimulate HIV-1 expression. These observations emphasize the dual role of NF-κB and AP-1 activations during HIV-1 cycle.

On the other hand, only little information is available concerning the expression of HIV uDNA and its regulation by cell-signaling pathways at the mechanistic level. First, it has been reported that HIV uDNA expression varies depending on the cell types^7^,^13^. Interestingly, uDNA gene expression is generally higher in non-proliferating cells (e.g., fully differentiate macrophages and memory resting T cells) as compared to proliferating cells, in part due to higher stability of uDNA in the absence of cell division^7^,^8^,^20^. Second, Kantor *et al*.^17^ have demonstrated that uDNA is organized in chromatin structures typical of silent chromatin that can be reactivated upon exposure to histone deacetylase (HDAC) inhibitors. Together, these findings suggest that gene expression from uDNA is effective and can be modulated, although the exact nature of uDNA forms involved (DNA_L_, 1-LTRc and/or 2-LTRc) remains elusive.

In this study, we characterized the expression of uDNA demonstrating for the first time that it is mainly supported by the expression from 1-LTRc and regulated in an opposite way, relatively to iDNA, following NF-κB pathway modulation. Using chromatin analyses and pharmacological treatments modulating the NF-κB pathway, we observed different patterns of binding of the NF-κB p50 and p65 subunits, Bcl-3, c-Fos and c-Jun to iDNA *vs* uDNA HIV LTR structured chromatin. Finally, we propose a model describing the interplay between transcriptional regulation mechanisms of iDNA and uDNA, highlighting the potential role of episomal forms in HIV-1 life cycle.

## Results

### Characterization of viral nucleic acid species supporting HIV transcription in the absence of integration

To gain new insights in the molecular mechanisms supporting HIV-1 expression in the absence of integration, we analyzed HIV expression in the presence of integrase strand transfer inhibitors (INSTIs) or D116N Class I mutation within the active site of the viral integrase. A parallel analysis of the impact of the three clinically used INSTIs (raltegravir, RAL; dolutegravir, DTG; elvitegravir, EVG) to avoid drug specific effects was also performed.

**Figure 1:**
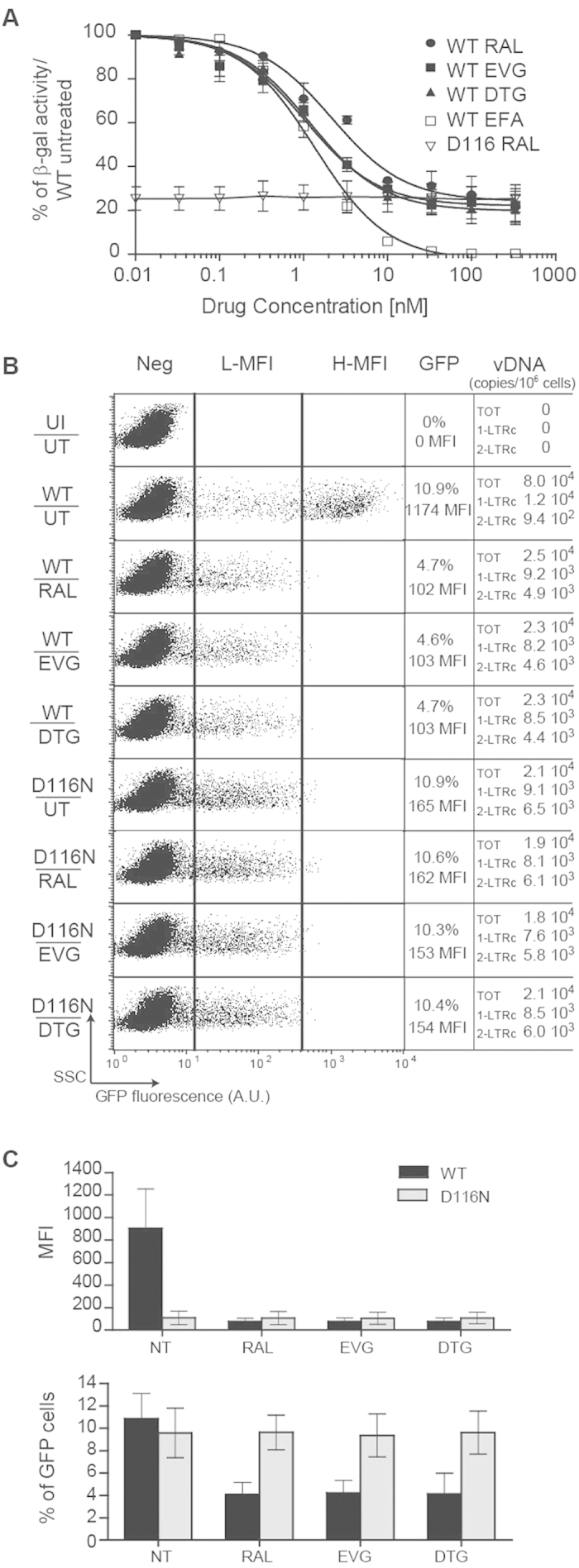
Expression of uDNA depending on DNA topology and integrase functionality. (**A**) Level of integrated HIV-1 LTR transactivation depending on Tat expression from integrated or unintegrated viral DNA. Hela-P4 cells were infected with HIV NL4-3 virus harboring a wild-type (WT) integrase or a catalytically defective integrase (“D116N”) (20 ng of p24^gag^ antigen on 10^6^ cells; ≈m.o.i. 0.2) in the presence of various concentrations of INSITs (raltegravir, RAL; dolutegravir, DTG; elvitegravir, EVG) or reverse transcriptase inhibitor (efavirenz, EFA). β-galactosidase expression was quantified 72 h post-infection by the CPRG method (see Methods for details). The results are presented as the percentage of reporter gene expression relative to the activity measured after infection by NL4.3 WT virus in untreated condition (without drug). Results were obtained from 3 independent experiments (mean ± s.e.m). (**B**,**C**) Cytometric analysis of viral transgene expression depending on viral integrase activity. MT4 T-cells were infected with either HIV-1 *env*^*–*^*gfp*^+^ harboring a wild-type (WT) integrase or HIV-1 with a catalytically defective integrase (“D116N”), each pseudotyped with VSV-G protein (4 ng of p24^gag^ antigen on 10^6^ cells; ≈m.o.i. 0.1), in the absence (UT) or presence of 200 nM RAL, DTG or EVG. Three days later, cytofluorometry was employed to determine the percentage and mean fluorescence intensity (MFI) of GFP expression. Dot plots show GFP fluorescence (expressed in arbitrary units, A.U.) depending on the Side-Scatter light (SSC). Quantitative-PCRs directed against viral DNAs (total, 1-LTRc and 2-LTRc) were analyzed 72 h post-infection. One representative experiment (panel (**B**)) and the results of 4 independent experiments (mean ± confidence intervals for a *p* value < 0.05 - panel (**C**)) are presented. Other abbreviations: H-MFI: high MFI, L-MFI: low MFI; Neg: negative cells; UI: uninfected condition; UT: untreated cells.

First, we explored viral expression through bulk analysis of HIV-1 expression in Hela-P4 reporter cells^35^. In this indirect reporter system, the transcription of the HIV-1 LTR-*lacZ* locus present in the Hela-P4 genome depends on transactivation by Tat proteins. We observed that, 3 days post-infection by the HIV-1 NL4-3 strain, the expression of the reporter gene decreased while the dose of INSTIs increased, until it reached a minimal threshold. Indeed, while integration was completely abolished, at concentrations above 100 nM (Figure S1), the reporter gene still represented ≈25% of the expression in the untreated condition (Fig. 1A), regardless of the INSTI compound used, in accordance with previous results obtained with RAL^36^. This minimal expression level was consistently observed during infection with NL4-3 virus expressing a D116N defective integrase, independently of the RAL doses (Fig. 1A). This latter observation indicates that the impact of RAL treatment on HIV expression is strictly due to the inhibition of integrase activity, with no side effect on HIV transcription. Finally, only efavirenz (EFA) fully inhibits HIV expression, confirming that reverse-transcription and expression from neosynthetized genomes are required for *lacZ* transcription in HelaP4 cells (Fig. 1A). Since this reporter gene expression is dependent upon the activation of the integrated HIV-1 LTR present in HelaP4 cell genome, these observations therefore indicate that Tat is expressed from unintegrated neosynthetized viral genomes at levels sufficient to transactivate integrated HIV-1 LTR.

**Figure 2:**
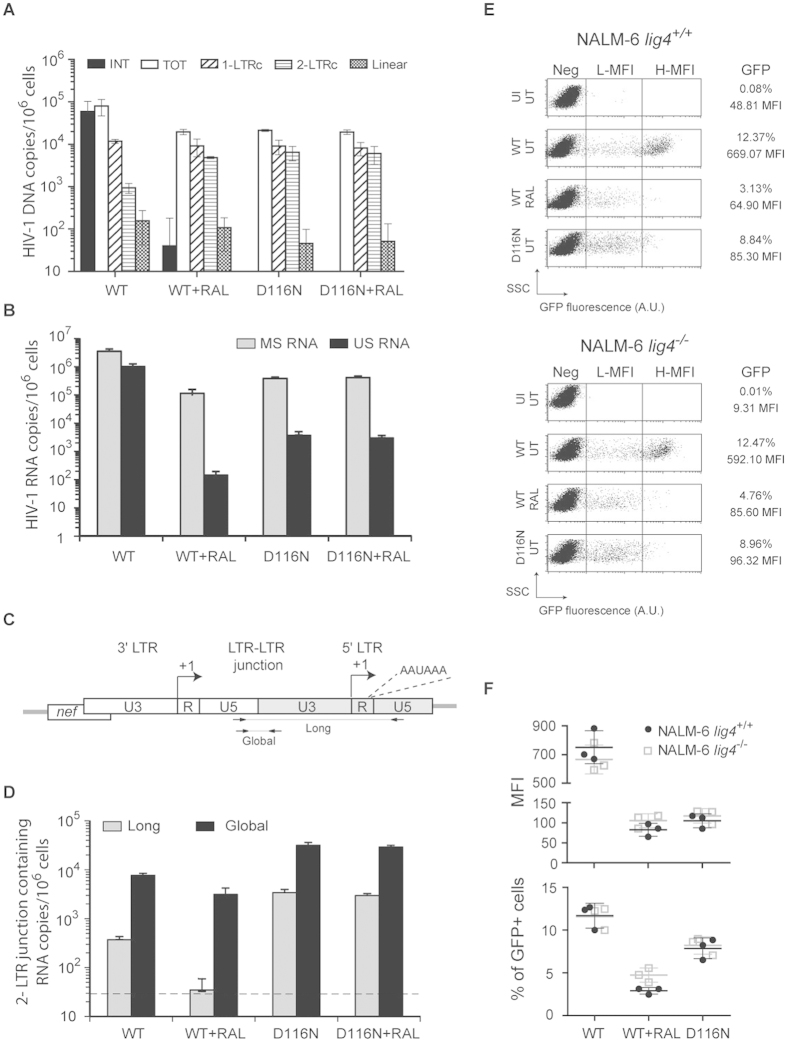
Expression of circular uDNAis mainly supported by 1-LTRc. (**A–D**) Quantification of RNA production from uDNA. MT4 T-cells were infected with HIV-1 *env*^*−*^*gfp*^+^ harboring either wild-type (WT) or a catalytically defective (“D116N”) integrase, each pseudotyped with VSV-G protein (≈m.o.i. 0.1), in the absence or presence of 200 nM RAL. Three days later, quantitative-PCR (qPCR) and RT-qPCR were performed to quantify viral DNA species (total, linear, 1-LTRc and 2-LTRc – panel (**A**)) along with unspliced (US, genomic viral RNA) and multi-spliced (MS, encoding accessory proteins) HIV-1 RNAs (panel (**B**)), and mRNAs containing the 2-LTRc junction (panel (**C,D**)). Primers and probes used to amplify long or total 2-LTR mRNAs are presented in panel (**C**). Of note, these RT-qPCRs allowed the specific quantification of mRNAs containing the 2-LTR junction, but did not quantify the entire expression from 2-LTRc given that transcripts can also be produced from the 5′-LTR (but are in this latter case undistinguishable from RNA produced from other HIV DNA templates). In panel (**D**), the grey line represents the detection threshold of long 2-LTR RNAs. The results are expressed in copies per million cells. They were obtained from 3 independent experiments (mean ± confidence intervals for a *p* value < 0.05). (**E**,**F**) Transgene expression depending on viral integrase and host ligase 4 activities. NALM-6 cells, proficient (*lig4*^+/+^) or deficient (*lig4*^−/−^) in ligase 4 activity, were infected with HIV-1 *env*^*−*^ *gfp*^+^ harboring either wild-type (WT) or a catalytically defective integrase (“D116N”), each pseudotyped with VSV-G protein (≈m.o.i. 0.1), in the absence (“UT”) or presence of 200 nM RAL (“RAL”). Three days later, cytofluorometry was employed to determine the percentage and mean fluorescence intensity (MFI) of GFP expression. Dot plots show GFP fluorescence (expressed in arbitrary units, A.U.) depending on the Side-Scatter light (SSC). One representative experiment (panel (**E**)) and the results of 3 independent experiments (barplots - panel (**F**)) are presented. Other abbreviations: H-MFI: high MFI, L-MFI: low MFI; Neg: negative cells; UI: uninfected cells.

To analyze HIV-1 uDNA expression at the single cell level, we performed a flow cytometric analysis of *gfp* transgene expression in T cells infected with recombinant reporter viruses, containing a *gfp-*IRES-*nef* cassette at the *nef* locus within HIV NL4–3 genome (“HIV-1 *env*^*−*^*gfp*^+^”, NLENG1-ES-IRES^15^). This highly sensitive method allows the direct analysis of viral expression in target cells after a single replication round. MT4 T-cells were infected with HIV-1 *env*^*−*^*gfp*^+^ viruses pseudopyted with VSV-G envelope, harboring functional (“WT”) or catalytically defective (“D116N”) integrases. The percentage of cells expressing the GFP transgene (% GFP^+^ cells) and their mean fluorescence intensity (MFI) were analyzed 3 days later. We observed that the population of highly fluorescent GFP^+^ cells (panel “H-MFI” in Fig. 1B) disappears in absence of integration, regardless of the experimental scheme (D116N *vs* INSTIs) used to abolish integrase activity. This observation confirms that integration is required to obtain high level of HIV-1 expression as previously observed by Gelderblom *et al*.^15^ using another INSTI (118-D-24). While integration inhibition reduced total transgene expression (% GFP^+^ cells x MFI) by 1 to 2 orders of magnitude, the percentage of cells expressing GFP remained constant or was only reduced by ≈2.5 fold after infection with D116N virus or with WT virus in presence of INSITs, respectively, whereas total viral DNA amounts were similar in these conditions (Figs 1B,C and 2A). These observations confirm that uDNA can sustain HIV-1 expression. Strikingly, the inactivation of the catalytic site of the HIV-1 integrase reduced the level of GFP expression but not the percentage of GFP^+^ cells (Fig. 1B,C), indicating that uDNA is expressed better in this condition than in presence of INSTIs and a functional integrase. Again, transgene expression after transduction with HIV-1 *env*^*−*^*gfp*^+^ D116N virus was insensitive to INSTIs (Fig. 1A–C). These observations were confirmed by the direct analysis of HIV-1 RNA copies per cell in these conditions (Fig. 2A–C). This suggests that INSTIs could form a complex with the WT integrase, but not with its D116N derivate, that may reduce uDNA expression. If the molecular mechanisms underlying a better expression from uDNA after infection by a virus harboring a defective integrase rather than after infection by a WT virus in presence of INSITs remain to be elucidated, it could be hypothesized that a complex formed between the WT integrase and INSITs could remain transiently on uDNA and so disturb transcription machinery activity.

To gain further insights on the nature of the uDNA species supporting HIV-1 expression, we quantified the different types of viral DNA genomes (total, linear, 1-LTRc, 2-LTRc) genomes individually as previously described^4^. Three days post-infection, linear viral DNA was detected in very small quantities in cells infected with HIV-1 *env*^*−*^*gfp*^+^ WT virus in the presence of RAL or with HIV-1 *env*^*−*^*gfp*^+^ D116N (Fig. 2A). This indicates that HIV-1 uDNA expression is mainly supported by circular genomes in these conditions.

Moreover, we observed that the ratio of HIV-1 unspliced RNA (US RNA, genomic RNA) to multispliced RNA (MS RNA) varies depending on integration (Fig. 2B): US RNAs are 2 to 3 log less abundant than MS RNAs in non-integrative conditions (WT virus^+^ RAL, D116N virus ± RAL), whereas it is only 1 log less abundant in the presence of integration. This suggests that integration can impact HIV-1 expression at the post-transcriptional level by direct or indirect mechanisms.

To explore the expression of 2-LTRc, we quantified the amount of RNAs containing the 2-LTRc junction (designated “global” in Fig. 2C) by RT-qPCR as previously described^37^. Of note, this technique only monitors the expression from the 3′LTR, the expression from the 5′ LTR being undistinguishable between the different HIV DNA species. In parallel, we developed a new RT-qPCR method (see Materiel & Methods) to analyze the level of viral RNA containing both the 2-LTR junction and the second polyA (AAUAAA) signal (designated “long” in Fig. 2C). It is noteworthy that less than 10% of the global 2-LTRc RNAs correspond to long 2-LTR RNAs, emphasizing transcription elongation impairment due to the presence of this termination signal. We observed that long 2-LTR junctions containing RNAs are 2 to 4 log less abundant than MS RNAs per cell after infection by HIV-1 *env*^*−*^*gfp*^+^ in all the conditions tested (Fig. 2B,C). Although the amount of RNA containing the 2-LTR junction only reflects the 2-LTRc transcription from the 3′LTR, these observations suggest that 2-LTRc may only poorly contribute to uDNA expression. Taking into account that ligase 4 is involved in the formation of 2-LTRc^38^, we further investigated the importance of 2-LTRc on HIV uDNA expression. We analyzed transgene expression after infection by HIV-1 *env*^*−*^*gfp*^+^ WT or D116N viruses of pre-B NALM-6 cells proficient or deficient in ligase 4 activity. We previously observed that NALM-6 *lig4*^*−/−*^ cells do not contain detectable levels of 2-LTRc, whereas 1-LTRc formation remains unaffected by *lig4* inactivation^4^. The absence of 2-LTRc does not significantly decrease transgene expression in NALM-6 *lig4*^*−/−*^ cells as compared to NALM-6 *lig4*^+*/*+^ cells (Fig. 2D–F). Thus, our results indicate that 3 days post-infection, HIV seems mainly expressed from 1-LTRc in the absence of integration.

### Opposite regulations of HIV transcription from uDNA and iDNA following pharmacological treatments modulating the NF-κB pathway

**Figure 3:**
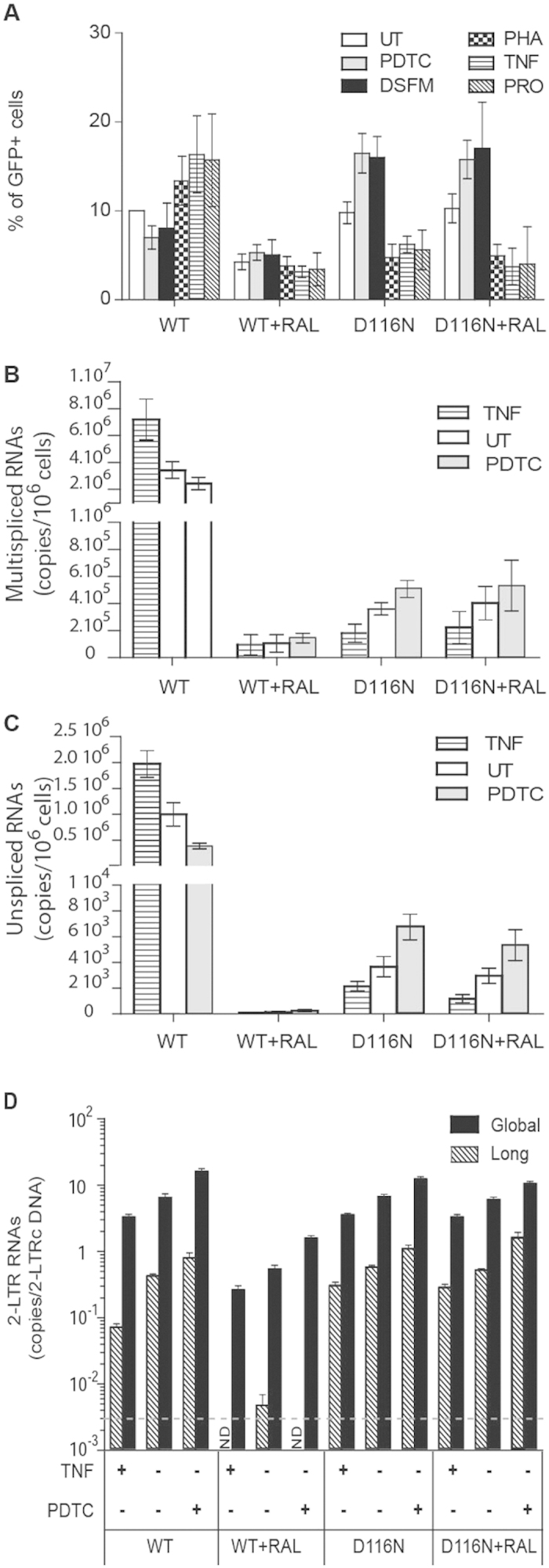
Opposite effects of NF-κB modulators on HIV-1 uDNA and iDNA transcriptional activities. (**A**) Impact of pharmacological treatments modulating the NF-κB pathway on HIV reporter gene expression depending on the viral integrative status. MT4 T-cells were infected with HIV-1 *env*^*−*^*gfp*^+^ harboring either wild-type (WT) integrase or a catalytically defective (“D116N”) integrase, each pseudotyped with VSV-G protein (4 ng of p24^gag^ antigen per 10^6^ cells; ≈m.o.i. 0.1). When indicated, 200 nM RAL was added 1 h before infection. Two days later, infected cells were incubated or not (UT) with pharmacological agents inhibiting the NF-κB pathway [50 μM pyrrolidinedithiocarbamate (PDTC), 10 μM disulfiram (DSFM)], or stimulating it [2.5 μg/mL phytohemagglutinin (PHA), 100 ng/mL tumor necrosis factor alpha (TNF), 5 μM prostratin (PRO)]. Three days post-infection, cytofluorometry was employed to determine the percentage of GFP-expressing cells. The results (mean ± confidence intervals for a *p* value < 0.05), were obtained from 3 to 7 independent experiments depending on the conditions. Treatments were maintained during the time of the experiment. (**B–D**) Impact of pharmacological treatments modulating the NF-KB pathway on HIV transcription depending on the viral integrative status. MT4 T-cells were treated and infected in the same conditions as described in panel A. Three days later, the amounts of unspliced, multi-spliced, long and global 2-LTR HIV-1 RNAs were determined by RT-qPCR. The efficiency of 2-LTRc transcription measured as the copy number of global or long 2-LTR RNAs per LTRc DNA template is presented in panel D. The grey line represents the detection threshold of long 2-LTR RNAs per 2-LTRc copy. The results (mean ± confidence intervals for a *p* value < 0.05 in panel **B** and **C**, mean ± s.e.m for panel **D**), were obtained from 3 to 6 independent experiments depending on the conditions. nd = not detected.

NF-κB is a key regulator of HIV transcription that can switch viral expression from latent to active state^39^. We thus investigated the impact of various pharmacological treatments modulating NF-κB activity on HIV-1 expression depending on the viral integrative status. MT4 T-cells were infected with HIV-1 *env*^*−*^*gfp*^+^ WT or D116N viruses pseudopyted with VSV-G envelope, in the presence or absence of the integrase inhibitor (RAL). Cells were incubated or not with pharmacological agents modulating NF-κB pathway. Tumor necrosis factor alpha (TNF-α), prostratin (PRO) and phytohemagglutinin (PHA) stimulate NF-κB pathway by the following mechanisms: TNF-α notably stimulates IκB (inhibitor of kappa B) phosphorylation through TRAF, RIP and/or MAP kinase pathway activation; PRO, a naturally occurring non-tumorigenic phorbol ester, activates protein kinase C and thereby stimulates IκB phosphorylation; PHA is a lectin known to activate the NF-κB pathway in T cells. Pyrrolidinedithiocarbamate (PDTC) and disulfiram (DSFM) inhibit the IκB kinase, thereby preventing NF-κB activation. To avoid any impact of these treatments on the early stages of the retroviral cycle (*e.g.* entry or viral DNA synthesis), they were applied 2 days post-infection. The percentages of cells expressing the GFP transgene (% GFP^+^ cells) and the HIV-1 RNA levels were analyzed 3 days post-infection. As expected, in condition allowing viral integration (conditions of infection by HIV-1 *env*^*−*^*gfp*^+^ WT virus in the absence of RAL), treatments stimulating the NF-κB pathway (i.e. TNF-α, PRO and PHA) promoted HIV-1 expression (Fig. 3). Strikingly, RAL treatment abolished the positive impact of TNF-α, PRO or PHA on HIV-1 *env*^*−*^*gfp*^+^ WT virus expression (Fig. 3A). Indeed, HIV-1 expression from uDNA after infection by the HIV-1 *env*^*−*^*gfp*^+^ D116N virus is strongly stimulated by treatments inhibiting NF-κB activation (PDTC and DSFM treatment conditions) while it is diminished by treatments stimulating NF-κB activation (TNF-α, PRO and PHA treatment conditions) (Fig. 3A). Notably, these opposite effects of pharmacological treatments were observed with US RNAs as well as with MS RNAs in all conditions tested (Fig. 3B,C).

The overall impact of NF-κB modulators was quantitatively more important when uDNA was expressed from HIV-1 *env*^*−*^*gfp*^+^ D116N virus (regardless of RAL treatment) than from HIV-1 *env*^*−*^*gfp*^+^ WT virus in the presence of RAL (Fig. 3). This observation reinforces the results of Fig. 1 indicating that the concomitant presence of RAL and a functional integrase catalytic triad is not optimal for uDNA expression. This suggested that the HIV-1 *env*^*−*^*gfp*^+^ D116N virus could constitute a better experimental model of HIV-1 uDNA expression in standard/native condition than HIV-1 *env*^*−*^*gfp*^+^ WT virus in the presence of RAL. To explore this hypothesis, we quantified 2-LTRs containing RNAs that represent the only quantifiable RNA unambiguously originating from uDNA expression, and more precisely from 2-LTRc. Their amounts after infection by integration-competent HIV-1 *env*^*−*^*gfp*^+^ virus (WT virus in absence of RAL) presented the same modulation profile by TNF-α and PDTC treatments as after infection by HIV-1 *env*^*−*^*gfp*^+^ D116N virus (Fig. 3D), reinforcing the interest of D116N viral model whose expression seems to reflect the regulation of integration-competent HIV-1 uDNA expression. After infection by HIV-1 *env*^*−*^*gfp*^+^ WT virus in presence of RAL, the efficiency of global 2-LTRc transcription (measured as the copy number of global 2-LTR RNAs per 2-LTRc template) was 1-log smaller than after infection by HIV-1 *env*^*−*^*gfp*^+^ WT virus in absence of RAL or by D116N virus (regardless of RAL treatment) (Fig. 3D). Moreover, the amount of long 2-LTR RNAs was at the limit of the detection threshold in this condition. Again, this suggests that after infection by HIV-1 *env*^*−*^*gfp*^+^ WT virus in presence of RAL, the presence of a native integrase associated to RAL may decrease further RNA polymerase initiation and processivity at the 2-LTR junction. This observation also comforts the poor contribution of long 2-LTR RNAs on uDNA expression from WT virus in presence of RAL. Furthermore, whereas HIV-1 protein expression levels are higher after infection by HIV-1 *env*^*−*^*gfp*^+^ WT than by HIV-1 *env*^*−*^*gfp*^+^ D116N viruses (Fig. 1B), the uDNA expression presents the same modulation profile in WT and D116N conditions, indicating that this modulation by the NF-κB pathway is independent of HIV-1 protein levels.

Taken together, these results indicate that HIV-1 transcription from iDNA and uDNA (mainly 1-LTRc) present opposite profile responses to pharmacological treatments modulating NF-κB activity.

### Differential transcription factor binding to HIV-1 uDNA and iDNA depend on the modulation of the NF-κB pathway

**Figure 4:**
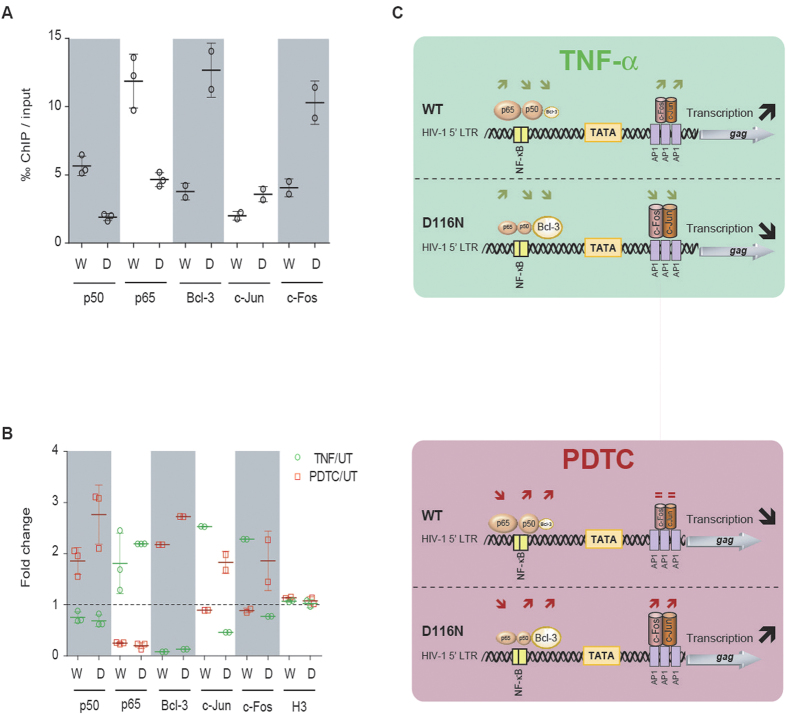
Differential binding of transcription factors to HIV-1 uDNA and iDNA depending on NF-κB pathway modulation. MT4 T-cells were infected with HIV-1 NL4-3 strains harboring a wild-type (“WT” or “W”) or a catalytically defective (“D116N” or “D”) integrase, each pseudotyped with VSV-G protein (100 ng of p24_gag_ antigen per 10^6^ cells; ≈m.o.i. 1). Two days later, the cells were incubated or not (UT) with pharmacological agents stimulating [100 ng/mL tumor necrosis factor alpha (TNF)] or inhibiting [50 μM pyrrolidinedithiocarbamate (PDTC)] the NF-κB pathway. Chromatin immunoprecipitation (ChIP) assays were performed on cellular extracts harvested 3 days post-infection, with antibodies directed against p50/NF-κB, p65/NF-κB, Bcl-3, c-Jun, c-Fos and H3 proteins. DNA obtained from the input or from the immunoprecipitated DNA were applied to qPCR performed on total HIV-1 DNA. The proportion of immunoprecipitated material per thousand (‰) of input material (normalized on immunoprecipitation yield) is presented in the panel A for p50, p65, Bcl-3, c-Jun and c-Fos ChIP assays. ChiP experiments performed with H3 antibodies led to the immunoprecipitation of 32–57% and 0.6–13% of input material in WT and D116N conditions, respectively. In panel (**B**), the results are expressed as the relative fold change of immunoprecipitated material in TNF-α or PDTC treated conditions compared to untreated condition. Schematic representations of ChIP and transcription results are presented in panel (**C**). Differences in the amount of proteins bound to WT *vs* D116N DNA (‰ of input) are represented by relatively smaller or bigger complexes, whereas significant variations after treatment (fold change) are indicated by arrows. The results were obtained from two independent experiments for Bcl-3, c-Jun and c-Fos and 3 independent experiments for NF-κB p65/p50 subunits.

To gain deeper insights on chromatin modification of HIV-1 DNA species depending on pharmacological treatments modulating the NF-κB pathway, chromatin immunoprecipitation (ChIP) assays were carried out on MT4 T-cells synchronously infected with HIV-1 NL4-3 WT or D116N strains, and incubated or not 2 days post-infection with TNF-α or PDTC. Three days post-infection, we analyzed the binding of the p65 and p50 subunits of NF-κB, c-Jun subunit of AP-1, c-Fos subunit of AP-1, and the Bcl-3 and H3 proteins to HIV-1 DNA in the R-U5 region (total viral DNA). As previously observed by Kantor *et al*.^17^, we observed that H3 is bound to uDNA and iDNA (see legend of Fig. 4), and so that episomal as well as integrated viral DNA is chromatinized. Under untreated conditions, all these transcription factors were detected in the HIV-1 R-U5 region after infection by both NL4-3 WT and D116N viruses (Fig. 4A). We observed significant bias in favor of p50/p65 binding to NL4-3 WT total DNA (=uDNA^+^ iDNA) *versus* Bcl-3 and AP-1 binding to NL4-3 D116N uDNA (Fig. 4A). This suggests that after infection by NL4-3 WT under standard conditions, p50/p65 is present on iDNA rather than on uDNA, whereas Bcl-3 and AP-1 are mainly bound to uDNA.

TNF-α treatment leads to increased binding of p65 to the HIV-1 R-U5 region after infection by both NL4-3 WT and D116N viruses (Fig. 4B,C). This increase correlates with the decreased binding of p50 and Bcl-3 proteins. Accordingly, the reverse profiles of transcription factor binding were observed after treatment with PDTC, with decreased amounts of p65 and increased amounts of Bcl-3 and p50 bound to NL4-3 WT and D116N DNAs (Fig. 4B,C). Interestingly, the AP-1 complex is the only tested transcription factor whose binding varies in opposite ways on NL4-3 WT and D116N DNAs depending on the pharmacological treatments applied: i) after TNF-α treatment, AP-1 binding to D116N uDNA decreased while binding to NL4-3 WT total DNAs increased; ii) reversely, after PDTC treatment, AP-1 binding to D116N uDNA increased but not to WT total DNAs (Fig. 4B,C). These results, associated with the observation that iDNA is the main HIV DNA species present within the cells 3 days after infection by NL4-3 WT (Figs 2A and 4), strongly suggest that AP-1 preferentially binds to iDNA and uDNA after TNF-α and PDTC treatments, respectively. This finding then places the AP-1 complex at the crossroad of the pathways controlling opposite profiles of transcription of iDNA and uDNA depending on pharmacological treatments modulating NF-κB activity.

Finally, treatments with IκB kinase inhibitors such as PDTC or DSFM correspond to optimal conditions for uDNA expression (Fig. 3). Moreover, PDTC also lead to the optimal binding of AP-1 and Bcl-3 to viral uDNA (≈7, 20 and 38‰ of the immunoprecipitated material with antibodies directed against c-Jun, c-Fos and Bcl-3, respectively; Fig. 4). On the other hand, we observed the maximal level of p65/NF-κB binding to viral iDNA (≈17‰ of the immunoprecipitated material with antibodies directed against p65/NF-κB; Fig. 4) in the condition that was optimal for NL4-3 WT virus expression, namely the TNF-α treatment. In conclusion, this study identifies for the first time opposite responses of HIV iDNA and uDNA expression to the NF-κB pathway modulation, correlated to differential binding of transcription factors to the HIV promoter depending on viral genome topology.

## Discussion

HIV-1 uDNA constitutes the most prevalent HIV-1 DNA species in CD4^+^ T cells and macrophages under antiretroviral treatments^40^. Data from this study are part of a bundle of evidences^7^,^8^,^12^,^13^,^14^,^15^,^16^,^17^,^18^,^19^ that reveal the potential role of HIV-1 uDNA in i) viral persistence, ii) cross-talk with HIV-1 iDNA, and iii) HIV-1 basal expression depending on cell type and activation state. Concerning the first aspect, we recently established that 2-LTRc can constitute a reservoir genome for *de novo* integration^16^. Cara *et al*.^12^ previously observed that HIV-1 expression is higher from 1-LTRc than from 2-LTRc synthetic circles of HIV-1 DNA mimicking uDNA. In the present study, we show that 2-LTRc DNA species only poorly contribute to HIV-1 expression in infected cells, and that 1-LTR circles constitute the main reservoir for HIV-1 expression in the absence of integration 3 days post-infection (Fig. 2).

We observed that the expression from the 3′LTR of 2-LTRc was poorly processive, with less than 10% of these RNAs that contain the second polyA (AAUAAA) signal (Fig. 2D). Indeed, Ashe *et al*.^41^ have shown that the downstream major splice donor site inhibits cleavage and polyadenylation at the 5′LTR promoter proximal site. This suggests that the recognition of this downstream major splice donor site may be impaired in the context of 2-LTRc genome.

We observed that the production of HIV-1 genomic RNAs was disfavored relatively to that of MS RNAs in the absence of integration as previously described in resting T cells^19^, suggesting that integration could have an impact on HIV-1 expression at the post-transcriptional level. This effect is often presumed to be indirect, for instance, due to the lower amounts of Rev proteins produced by infected cells. It may also result from a difference at the transcriptional level, due to transcription from an internal promoter^42^,^43^, allowing the transcription of MS RNAs but not of the genomic RNA. Both of these mechanisms could contribute to the requirement of HIV-1 integration for robust viral production. However, HIV-1 particle production is still possible in the absence of integration, as recently reported^17^,^19^.

Here, we report that uDNA can even influence the expression of latent iDNA as Tat expressed from neosynthesized viral uDNA can transactivate iDNA HIV-1 LTR in HeLa-P4 (Fig. 1A). This opens new insights on the possible interplay between uDNA and iDNA at the transcriptional level, in addition to the cross-talk at the genomic level through recombination^15^,^17^.

Cell type and status could impact on HIV-1 uDNA expression through differences in proliferation rates or by the availability of transcription factors modulating viral expression. Indeed, HIV transcription is controlled by both host transcription factor mobilization and chromatin decondensation. Kantor *et al*.^17^ have demonstrated that uDNA HIV-1 genomes are organized in nucleosomal structures, enriched in histone modifications typical of silenced chromatin that can be reactivated by HDAC inhibitor treatments. Here, we have explored the impact of pharmacological treatments modulating the state of transcription factor activation on HIV-1 uDNA expression. Unexpectedly, we reveal that HIV-1 iDNA and uDNA present opposite patterns of response to components modulating NF-κB treatment (Figs 3 and 4). Recently, Chan and collaborators also reported that gene expression from uDNA was somewhat regulated differently from iDNA in response to various pharmacological treatments of resting T-cells, uDNA responses displaying a wider dynamic range^19^. In MT-4 cells, at the molecular level, and in presence of integration, HIV-1 expression is positively correlated to the binding of NF-κB p65-p50 heterodimer to viral DNA, with a positive and negative impact of TNF-α and PDTC treatments, respectively. Conversely, in the absence of integration, HIV-1 expression is maximum under PDTC treatment with increased binding of AP-1 and Bcl-3 to viral uDNA. Moreover, the Bcl-3 protein is more prevalent on viral DNA in the absence than in the presence of integration. It could be hypothesized that differences in chromatin topological constraints (twist/coil/roll) between iDNA and uDNA may contribute to differential binding of transcription factors to their binding sites located within a highly conserved nucleosome sequence (namely “nuc-1”). Of note, Bcl3 expression varies depending on the cell type and environment, this protein being expressed at a significant level in MT-4 cells^44^. This suggests that Bcl-3 cell content could modulate the impact of pharmacological treatments on HIV-1 expression. Given that interaction of the NF-κB p50-p50 homodimer with Bcl-3 can switch the balance from inhibition to activation of HIV transcription^25^,^26^,^27^, we propose that HIV-1 uDNA expression may be controlled by the Bcl3-p50-p50 rather than by the NF-κB p65-p50 heterodimer. This interaction may be particularly important to consider in differentiated macrophages whose constitutive nuclear pool of NF-κB is mostly composed of p50^28^, emphasizing the potential role of uDNA in the pathophysiology of HIV associated with the persistence and dissemination due to macrophages.

In this study, AP-1 appeared as the only transcription factor that presents opposite patterns of HIV-1 DNA binding variation depending on integration and pharmacological treatment modulating at least the NF-κB pathway: AP-1 binding increases on viral iDNA and uDNA in the presence of TNF-α and PDTC, respectively. Therefore, AP-1 binding is positively correlated with HIV-1 expression independently of integration and of the composition of the NF-κB complex (being either the p65-p50 heterodimer in the presence of integration, or Bcl-3-p50-p50 in the absence of integration). Indeed, NF-κB and AP-1 transcription factors are regulated by distinct mechanisms but appear to be connected and activated simultaneously by several stimuli notably through MAPK pathway activation^45^. Moreover, NF-κB and AP-1 transcription factors can cooperatively regulate the HIV-1 promoter^24^. Our study confirms that AP-1 could contribute to HIV emergence from latency.

Viral activation in latently infected cells represents a strategy to purge HIV-1 reservoirs. Indeed, promising approaches based on the use of potent latency-reversing agents of which agonists of the protein kinase C (such as prostratin) have been reported^46^,^47^. Interestingly, our study highlights a potential limit of pharmacological treatments stimulating of NF-κB pathway given that the expression of uDNA is weakened by prostratin in MT-4 cell model (Fig. 3A), leaving a viral reservoir that can potentially lead to clinical relapse. Thus, we report observations that differentiate viral reservoirs from post-integration latency and the reservoirs from uDNA containing pre-integration latency, based on their molecular mechanisms of reactivation.

Finally, we propose a model describing the transcriptional mechanisms of iDNA and uDNA highlighting that HIV-1 uDNA can extend viral expression to situations in which the NF-κB pathway is down-stimulated.

## Methods

### Cells and viruses

Human HEK293T (ATCC CRL-11268) and HeLa-P4 cells^35^ were maintained in Dulbecco’s Modified Eagle Medium (DMEM). MT-4 cells^48^, NALM-6 (B cell precursor leukemia) and its derivative deficient in ligase 4 activity (NALM-6 *ligase4*^*−/−*^ 49 also known as “N114P2”^50^ or “NALM-114”^4^) were cultured in RPMI-I640 medium. All media, purchased from Gibco (Life Technologies Co.), were supplemented with 10% fetal bovine serum (PAA Laboratories GmbH, Pasching, Austria) and 1% penicillin/streptomycin (100 units/mL). All cell lines used here were incubated at 37 °C, under 5% CO_2_ atmosphere. HIV-1 stocks were prepared by calcium phosphate-mediated transfection of HEK293T cells, as previously described^51^, with shuttle vector plasmids encoding HIV-1 NL4-3 (GenBank: AF324493.1), NLENG1-ES-IRES-WT or NLENG1-ES-IRES-D116N^15^. The two latter vectors originate from HIV NL4-3 strain, contain a *gfp-*IRES-*nef* cassette at the *nef* locus, and encode a wild-type or a catalytically defective integrase. For simplicity, we designate these HIV-1 *env*^*−*^*gfp*^+^ vectors here as “WT” or “D116N”. The HIV-1 *env*^*−*^*gfp*^+^ vector or NL4-3 native virus (only when used for ChIP assays) were pseudotyped by cotransfecting HEK293T cells with the shuttle plasmid and the pMD.G plasmid encoding a VSV-G envelope^52^. Virus preparations were treated with DNase I (Takara Bio, CA) in the presence of 10 mM MgCl_2_ at 37 °C for 30 min and ultracentrifuged (17,000 g for 1 h at 4 °C). Viruses were finally resuspended in PBS 1X at a concentration ratio of 200 and stored at −80 °C.

### Viral infections and treatments

The HIV-1 p24^gag^ antigen contents in viral inocula were determined by enzyme-linked immunosorbent assay (Perkin-Elmer Life Sciences). To obtain a multiplicity of infection (m.o.i.) reaching 0.1–0.2, 20 ng or 4 ng of p24^gag^ antigen per 10^6^ cells was used for infection with pNL4-3 virus and HIV-1 *env*^*−*^*gfp*^+^ VSV-G pseudotyped particles, respectively. Three hours post-infection, the cells were washed three times with PBS 1X. When required, integrase inhibitors (raltegravir, RAL; dolutegravir, DTG; elvitegravir, EVG), or reverse trancriptase inhibitor (Efavirenz, EFA) were added in the culture medium 1 h before infection. The dose-response curves were calculated by standard nonlinear regression using a sigmoidal dose-response equation (GraphPad Prism). When required, 2 days post-infection, MT4 T-cells were incubated with pharmacological agents modulating the NF-κB pathway: pyrrolidinedithiocarbamate (PDTC, 50 μM), disulfiram (DSFM, 10 μM), or phytohemagglutinin (PHA, 2.5 μg/mL), tumor necrosis factor alpha (TNF, 100 ng/mL), prostratin (PRO, 5 μM).

### HIV infectivity assay

The single-cycle titers of the NL4-3 virus was determined in HeLa-P4 cells by the CPRG method as previously described^36^. These are HeLa CD4 LTR-*lacZ* cells in which *lacZ* expression is induced by the HIV transactivator Tat, allowing precise quantification of HIV-1 infectivity. For the HIV-1 *env*^*−*^*gfp*^+^ vectors, transgene expression [*i.e*., the percentage and geometric mean of fluorescence intensity (MFI) of GFP^+^ cells] was estimated by flow cytometry using a FACSCalibur^TM^ cytofluorometer (BD Biosciences). The functional m.o.i. was estimated by transduction of MT-4 cells with serial dilutions of viruses, and cytometric analysis of GFP-expressing cells (72 h post-transduction). The linear phase of transduction (≈3–30% of GFP^+^ cells) was used to calculate the apparent m.o.i.

### Quantifications of HIV-1 DNAs and RNAs

Two to five million cells were collected at each time point. They were washed in PBS 1X, and dry cell pellets were frozen at −20 °C until used. DNA and RNA from infected cells was purified with QIAamp DNA blood mini kit (QIAGEN) and RNeasy® Plus Mini Kit (QIAGEN), respectively, according to the manufacturer’s instructions. Quantifications of RNA species were performed by real-time one-step RT-PCR with a Light Cycler instrument (Roche Life Science). Accordingly, all DNA quantifications were performed by real time PCR (“qPCR”) in the same apparatus. They were performed on nucleic acid amounts equivalent to ≈200,000 cells. Sequences of primers and probes used for qPCR for total HIV-1, 2-LTRc and integrated viral DNA absolute quantifications have been described previously^5^. 1-LTRc and linear HIV DNA (DNA_L_) were quantified according to^4^. Copy numbers of total HIV-1 DNA, 2-LTRc and DNA_L_ were determined from calibration curves obtained by amplifying pre-determined quantities of cloned DNA with matching sequences ranging from 10 to 10^5^ copies. The iDNA copy number was determined from a calibration curve obtained by concomitant two-stage PCR amplification of serial dilutions of an iDNA standard (from HeLa R7 Neo^5^) mixed with uninfected cell DNA to yield 50,000 cell equivalents. The number of cell equivalents in sample DNA was calculated by amplifying the gene encoding β-globin^53^. Multispliced (MS RNAs), unspliced (US RNAs) and global 2-LTR HIV-1 RNAs were quantified as previously described^37^. Long 2-LTR-RNAs were quantified using the NuTaq-2-for (5′-TCAGACCCTTTTAGTCAGTGTGGA-3′) and MH531-rev (5′-ACACAACAGACGGGCACACA-3′) primers, associated with HIV-FL (5′-CCACACACAAGGCTACTTCCCTGA-3′) and HIV-LC (5′-TGGCAGAACTACACACCAGGGC-3′) probes. RT-PCR cycle conditions were: reverse transcription (61 °C, 35 min); denaturation (95 °C, 8 min); 15 cycles of (95 °C, 10 s; 60 °C, 10 s; 72 °C, 20 s) and cycles with annealing temperature decreasing by 0.5 °C/cycle until reaching 59 °C.

### Chromatin immunoprecipitation (ChIP)

ChIP assays were performed as previously described^16^,^54^. Briefly, 72 h post-infection, 10^7^ (for the study of transcription factor binding) or 10^6^ (for the histone control) infected MT4 cells were treated with 1% formaldehyde for 10 min at 37 °C. Subsequent procedures were performed on ice with protease inhibitors. Cross-linked cells were harvested, washed with PBS, and lysed in lysis buffer (1% SDS, 10 mM EDTA, 50 mM Tris–HCl, pH = 8.1) for 10 min at 4 °C. Chromatin was sonicated (six 10 s pulses at an amplitude of 30%). After centrifugation (14,000 g, 10 min, 4 °C), the supernatant was diluted 10-fold with ChIP dilution buffer (0.01% SDS, 1% Triton X-100, 1.2 mM EDTA, 16.7 mM Tris–HCl, pH = 8.1, 167 mM NaCl). Diluted extracts were pre-cleared with salmon sperm DNA-protein A-agarose beads (ChIP assay kit, Upstate). One tenth of the diluted extract was kept for qPCR (input). The remaining extracts were incubated for 16 h at 4 °C with 1 μg/ml of the specific antibody from Upstate Biotechnology (anti-histone H3, p50/NF-kB, p65/NF-kB) or from Santa Cruz (Bcl-3, c-Jun and c-Fos) and then further incubated for 1 h with salmon sperm DNA-protein A-agarose beads. Following extensive washing, bound DNA fragments were eluted. DNA was recovered by incubation for 4 h at 65 °C in elution buffer supplemented with 200 mM NaCl and incubated with proteinase-K (20 μg/ml) for 1 h at 45 °C. DNA was extracted before PCR quantification. The immunoprecipitated and input DNA were subjected to PCR quantification. The proportion of immunoprecipitated material was normalized on immunoprecipitation yield. The results are expressed as the fraction of immunoprecipitated DNA for each set of conditions (% of input) and as fold increase compared to untreated cells (fold change).

### Statistical procedure

Data and error bars (standard deviation, standard error of the mean and the confidence interval for a *p* value of 0.05) were analyzed and calculated using Microsoft Excel (Microsoft, Redmond, WA) and Prism (GraphPad Software; La Jolla, CA).

## Additional Information

**How to cite this article**: Thierry, S. *et al*. Opposite transcriptional regulation of integrated *vs* unintegrated HIV genomes by the NF-κB pathway. *Sci. Rep.* **6**, 25678; doi: 10.1038/srep25678 (2016).

## Supplementary Material

Supplementary Information

## References

[b1] Bukrinsky M. I., Stanwick T. L., Dempsey M. P. & Stevenson M. Quiescent T lymphocytes as an inducible virus reservoir in HIV-1 infection. Science 254, 423–427 (1991). 1925601 10.1126/science.1925601 PMC9524215

[b2] Chun T. W. . Presence of an inducible HIV-1 latent reservoir during highly active antiretroviral therapy. Proceedings of the National Academy of Sciences of the United States of America 94, 13193–13197 (1997). 9371822 10.1073/pnas.94.24.13193 PMC24285

[b3] Butler S. L., Johnson E. P. & Bushman F. D. Human immunodeficiency virus cDNA metabolism: notable stability of two-long terminal repeat circles. Journal of virology 76, 3739–3747 (2002). 11907213 10.1128/JVI.76.8.3739-3747.2002 PMC136088

[b4] Munir S., Thierry S., Subra F., Deprez E. & Delelis O. Quantitative analysis of the time-course of viral DNA forms during the HIV-1 life cycle. Retrovirology 10, 87, doi: 10.1186/1742-4690-10-87 (2013). 23938039 PMC3766001

[b5] Brussel A. & Sonigo P. Analysis of early human immunodeficiency virus type 1 DNA synthesis by use of a new sensitive assay for quantifying integrated provirus. Journal of virology 77, 10119–10124 (2003). 12941923 10.1128/JVI.77.18.10119-10124.2003 PMC224570

[b6] Sharkey M. E. . Persistence of episomal HIV-1 infection intermediates in patients on highly active anti-retroviral therapy. Nat Med 6, 76–81, doi: 10.1038/71569 (2000). 10613828 PMC9513718

[b7] Gillim-Ross L., Cara A. & Klotman M. E. Nef expressed from human immunodeficiency virus type 1 extrachromosomal DNA downregulates CD4 on primary CD4^+^ T lymphocytes: implications for integrase inhibitors. J Gen Virol 86, 765–771, doi: 10.1099/vir.0.80570-0 (2005). 15722538

[b8] Kelly J. . Human macrophages support persistent transcription from unintegrated HIV-1 DNA. Virology 372, 300–312, doi: 10.1016/j.virol.2007.11.007 (2008). 18054979 PMC2276161

[b9] Pace M. J., Graf E. H. & O’Doherty U. HIV 2-long terminal repeat circular DNA is stable in primary CD4^+^ T Cells. Virology 441, 18–21, doi: 10.1016/j.virol.2013.02.028 (2013). 23537959 PMC3983779

[b10] Graf E. H. . Elite suppressors harbor low levels of integrated HIV DNA and high levels of 2-LTR circular HIV DNA compared to HIV^+^ patients on and off HAART. PLos Pathog 7, e1001300, doi: 10.1371/journal.ppat.1001300 (2011). 21383972 PMC3044690

[b11] Buzon M. J. . Inhibition of HIV-1 integration in ex vivo-infected CD4 T cells from elite controllers. Journal of virology 85, 9646–9650, doi: 10.1128/JVI.05327-11 (2011). 21734042 PMC3165766

[b12] Cara A., Cereseto A., Lori F. & Reitz M. S. Jr. HIV-1 protein expression from synthetic circles of DNA mimicking the extrachromosomal forms of viral DNA. The Journal of biological chemistry 271, 5393–5397 (1996). 8621393 10.1074/jbc.271.10.5393

[b13] Nakajima N., Lu R. & Engelman A. Human immunodeficiency virus type 1 replication in the absence of integrase-mediated dna recombination: definition of permissive and nonpermissive T-cell lines. Journal of virology 75, 7944–7955 (2001). 11483739 10.1128/JVI.75.17.7944-7955.2001 PMC115038

[b14] Poon B., Chang M. A. & Chen I. S. Vpr is required for efficient Nef expression from unintegrated human immunodeficiency virus type 1 DNA. Journal of virology 81, 10515–10523, doi: 10.1128/JVI.00947-07 (2007). 17652391 PMC2045493

[b15] Gelderblom H. C. . Viral complementation allows HIV-1 replication without integration. Retrovirology 5, 60, doi: 10.1186/1742-4690-5-60 (2008). 18613957 PMC2474848

[b16] Thierry S. . Integrase inhibitor reversal dynamics indicate unintegrated HIV-1 dna initiate de novo integration. Retrovirology 12, 24, doi: 10.1186/s12977-015-0153-9 (2015). 25808736 PMC4372172

[b17] Kantor B., Ma H., Webster-Cyriaque J., Monahan P. E. & Kafri T. Epigenetic activation of unintegrated HIV-1 genomes by gut-associated short chain fatty acids and its implications for HIV infection. Proceedings of the National Academy of Sciences of the United States of America 106, 18786–18791, doi: 10.1073/pnas.0905859106 (2009). 19843699 PMC2773968

[b18] Shimura K., Miyazato P., Oishi S., Fujii N. & Matsuoka M. Impact of HIV-1 infection pathways on susceptibility to antiviral drugs and on virus spread. Virology 484, 364–376, doi: 10.1016/j.virol.2015.06.029 (2015). 26186575

[b19] Chan C. N. . HIV-1 latency and virus production from unintegrated genomes following direct infection of resting CD4 T cells. Retrovirology 13, 1, doi: 10.1186/s12977-015-0234-9 (2016). 26728316 PMC4700562

[b20] Sloan R. D. & Wainberg M. A. The role of unintegrated DNA in HIV infection. Retrovirology 8, 52, doi: 10.1186/1742-4690-8-52 (2011). 21722380 PMC3148978

[b21] Van Lint C., Bouchat S. & Marcello A. HIV-1 transcription and latency: an update. Retrovirology 10, 67, doi: 10.1186/1742-4690-10-67 (2013). 23803414 PMC3699421

[b22] Turrini F. . HIV-1 transcriptional silencing caused by TRIM22 inhibition of Sp1 binding to the viral promoter. Retrovirology 12, 104, doi: 10.1186/s12977-015-0230-0 (2015). 26683615 PMC4683785

[b23] Colin L. & Van Lint C. Molecular control of HIV-1 postintegration latency: implications for the development of new therapeutic strategies. Retrovirology 6, 111, doi: 10.1186/1742-4690-6-111 (2009). 19961595 PMC2797771

[b24] Kilareski E. M., Shah S., Nonnemacher M. R. & Wigdahl B. Regulation of HIV-1 transcription in cells of the monocyte-macrophage lineage. Retrovirology 6, 118, doi: 10.1186/1742-4690-6-118 (2009). 20030845 PMC2805609

[b25] Franzoso G. . The candidate oncoprotein Bcl-3 is an antagonist of p50/NF-kappa B-mediated inhibition. Nature 359, 339–342, doi: 10.1038/359339a0 (1992). 1406939

[b26] Zhang M. Y., Harhaj E. W., Bell L., Sun S. C. & Miller B. A. Bcl-3 expression and nuclear translocation are induced by granulocyte-macrophage colony-stimulating factor and erythropoietin in proliferating human erythroid precursors. Blood 92, 1225–1234 (1998). 9694711

[b27] Dechend R. . The Bcl-3 oncoprotein acts as a bridging factor between NF-kappaB/Rel and nuclear co-regulators. Oncogene 18, 3316–3323, doi: 10.1038/sj.onc.1202717 (1999). 10362352

[b28] Asin S., Bren G. D., Carmona E. M., Solan N. J. & Paya C. V. NF-kappaB cis-acting motifs of the human immunodeficiency virus (HIV) long terminal repeat regulate HIV transcription in human macrophages. Journal of virology 75, 11408–11416, doi: 10.1128/JVI.75.23.11408-11416.2001 (2001). 11689622 PMC114727

[b29] Pertel T. . TRIM5 is an innate immune sensor for the retrovirus capsid lattice. Nature 472, 361–365, doi: 10.1038/nature09976 (2011). 21512573 PMC3081621

[b30] Galao R. P., Le Tortorec A., Pickering S., Kueck T. & Neil S. J. Innate sensing of HIV-1 assembly by Tetherin induces NFkappaB-dependent proinflammatory responses. Cell host & microbe 12, 633–644, doi: 10.1016/j.chom.2012.10.007 (2012). 23159053 PMC3556742

[b31] Tokarev A. . Stimulation of NF-kappaB activity by the HIV restriction factor BST2. Journal of virology 87, 2046–2057, doi: 10.1128/JVI.02272-12 (2013). 23221546 PMC3571454

[b32] Lascano J., Uchil P. D., Mothes W. & Luban J. TRIM5 Retroviral Restriction Activity Correlates with the Ability To Induce Innate Immune Signaling. Journal of virology 90, 308–316, doi: 10.1128/JVI.02496-15 (2015). 26468522 PMC4702541

[b33] Postler T. S. & Desrosiers R. C. The cytoplasmic domain of the HIV-1 glycoprotein gp41 induces NF-kappaB activation through TGF-beta-activated kinase 1. Cell host & microbe 11, 181–193, doi: 10.1016/j.chom.2011.12.005 (2012). 22341466 PMC3285415

[b34] Liu R. . HIV-1 Vpr stimulates NF-kappaB and AP-1 signaling by activating TAK1. Retrovirology 11, 45, doi: 10.1186/1742-4690-11-45 (2014). 24912525 PMC4057933

[b35] Charneau P., Alizon M. & Clavel F. A second origin of DNA plus-strand synthesis is required for optimal human immunodeficiency virus replication. Journal of virology 66, 2814–2820 (1992). 1560526 10.1128/jvi.66.5.2814-2820.1992 PMC241038

[b36] Delelis O. . Impact of Y143 HIV-1 integrase mutations on resistance to raltegravir *in vitro* and *in vivo*. Antimicrob Agents Chemother 54, 491–501, doi: 10.1128/AAC.01075-09 (2010). 19901095 PMC2798554

[b37] Brussel A. & Sonigo P. Evidence for gene expression by unintegrated human immunodeficiency virus type 1 DNA species. Journal of virology 78, 11263–11271, doi: 10.1128/JVI.78.20.11263-11271.2004 (2004). 15452245 PMC521838

[b38] Li L. . Role of the non-homologous DNA end joining pathway in the early steps of retroviral infection. The EMBO journal 20, 3272–3281, doi: 10.1093/emboj/20.12.3272 (2001). 11406603 PMC150207

[b39] Nabel G. & Baltimore D. An inducible transcription factor activates expression of human immunodeficiency virus in T cells. Nature 326, 711–713, doi: 10.1038/326711a0 (1987). 3031512

[b40] Chun T. W. . Quantification of latent tissue reservoirs and total body viral load in HIV-1 infection. Nature 387, 183–188, doi: 10.1038/387183a0 (1997). 9144289

[b41] Ashe M. P., Griffin P., James W. & Proudfoot N. J. Poly(A) site selection in the HIV-1 provirus: inhibition of promoter-proximal polyadenylation by the downstream major splice donor site. Genes Dev 9, 3008–3025 (1995). 7498796 10.1101/gad.9.23.3008

[b42] Verdin E. & Van Lint C. Internal transcriptional regulatory elements in HIV-1 and other retroviruses. Cell Mol Biol (Noisy-le-grand) 41, 365–369 (1995). 7580829

[b43] Colin L. . The AP-1 binding sites located in the pol gene intragenic regulatory region of HIV-1 are important for viral replication. Plos one 6, e19084, doi: 10.1371/journal.pone.0019084 (2011). 21526160 PMC3079759

[b44] Saito K., Saito M., Taniura N., Okuwa T. & Ohara Y. Activation of the PI3K-Akt pathway by human T cell leukemia virus type 1 (HTLV-1) oncoprotein Tax increases Bcl3 expression, which is associated with enhanced growth of HTLV-1-infected T cells. Virology 403, 173–180, doi: 10.1016/j.virol.2010.04.018 (2010). 20471052

[b45] Fujioka S. . NF-kappaB and AP-1 connection: mechanism of NF-kappaB-dependent regulation of AP-1 activity. Mol Cell Biol 24, 7806–7819, doi: 10.1128/MCB.24.17.7806-7819.2004 (2004). 15314185 PMC507000

[b46] Jiang G. . Reactivation of HIV latency by a newly modified Ingenol derivative via protein kinase Cdelta-NF-kappaB signaling. AIDS 28, 1555–1566, doi: 10.1097/QAD.0000000000000289 (2014). 24804860 PMC4922310

[b47] Darcis G. . An In-Depth Comparison of Latency-Reversing Agent Combinations in Various *In Vitro* and Ex Vivo HIV-1 Latency Models Identified Bryostatin-1 + JQ1 and Ingenol-B + JQ1 to Potently Reactivate Viral Gene Expression. PLoS Pathog 11, e1005063, doi: 10.1371/journal.ppat.1005063 (2015). 26225566 PMC4520688

[b48] Foley G. E. . Continuous Culture of Human Lymphoblasts from Peripheral Blood of a Child with Acute Leukemia. Cancer 18, 522–529 (1965). 14278051 10.1002/1097-0142(196504)18:4<522::aid-cncr2820180418>3.0.co;2-j

[b49] Grawunder U., Zimmer D., Fugmann S., Schwarz K. & Lieber M. R. DNA ligase IV is essential for V(D)J recombination and DNA double-strand break repair in human precursor lymphocytes. Mol Cell 2, 477–484 (1998). 9809069 10.1016/s1097-2765(00)80147-1

[b50] Smith J. . Impact of DNA ligase IV on the fidelity of end joining in human cells. Nucleic acids research 31, 2157–2167 (2003). 12682366 10.1093/nar/gkg317 PMC153745

[b51] Manic G. . 3′ self-inactivating long terminal repeat inserts for the modulation of transgene expression from lentiviral vectors. Human gene therapy methods 23, 84–97, doi: 10.1089/hgtb.2011.154 (2012). 22456436

[b52] Ory D. S., Neugeboren B. A. & Mulligan R. C. A stable human-derived packaging cell line for production of high titer retrovirus/vesicular stomatitis virus G pseudotypes. Proceedings of the National Academy of Sciences of the United States of America 93, 11400–11406 (1996). 8876147 10.1073/pnas.93.21.11400 PMC38069

[b53] Manic G. . Impact of the Ku complex on HIV-1 expression and latency. PloS one 8, e69691, doi: 10.1371/journal.pone.0069691 (2013). 23922776 PMC3726783

[b54] Thierry S. . Cell cycle arrest in G2 induces human immunodeficiency virus type 1 transcriptional activation through histone acetylation and recruitment of CBP, NF-kappaB, and c-Jun to the long terminal repeat promoter. Journal of virology 78, 12198–12206, doi: 10.1128/JVI.78.22.12198-12206.2004 (2004). 15507606 PMC525107

