# Supplementary material for: Opposite transcriptional regulation of integrated vs unintegrated HIV genomes by the NF-κB pathway
Source: Sci Rep. 2016 May 11;6:25678. doi: 10.1038/srep25678 (PMC4863372; doi:10.1038/srep25678)
Supplement: Supplementary Information [file srep25678-s1.pdf]

Supplementary information

TITLE

Opposite transcriptional regulation of integrated *vs* unintegrated HIV genomes by the NF-κB pathway

Sylvain Thierry[1,2], Eloïse Thierry[1,3], Frédéric Subra[1], Eric Deprez[1], Hervé Leh[1], Stéphanie Bury-Moné[1,*], Olivier Delelis[1,*]

[1]LBPA, ENS Cachan, CNRS UMR8113, IDA FR3242, Université Paris-Saclay, F-94235 Cachan, France

[2]Present address: CNRS UMR3347 Institut Curie, Centre Universitaire d'Orsay, Paris-Sud, 91405 Orsay, France

[3]Present address: CIRI, INSERM U1111, CNRS UMR5308, Université Claude Bernard Lyon-1, Ecole Normale Supérieure de Lyon, Lyon 69007, France

*These authors contributed equally to this work. Email: delelis@lbpa.ens-cachan.fr; stephanie.bury-mone@lbpa.ens-cachan.fr

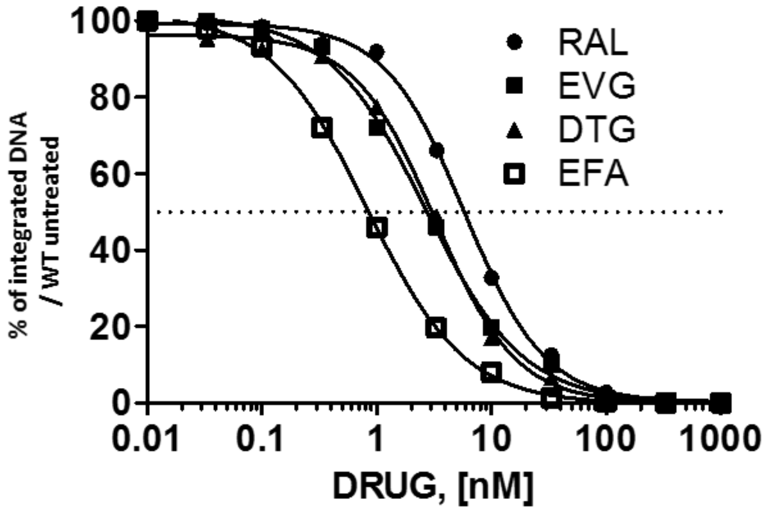

Supplementary Figure 1: **Dose-response inhibition of HIV integration by latest generations of INSITs**

MT4 T-cells were infected with HIV-1 NL4.3 strain (20 ng of $p24_{gag}$ antigen on 10^6 cells; ≈ m.o.i. 0.1) in presence of various concentrations of INSITs (raltegravir, RAL; dolutegravir, DTG; elvitegravir, EVG). Incubation with a reverse-transcriptase inhibitor (Efavirenz, EFA) was used as a control condition. Integrated HIV-1 DNA was quantified 72h post-infection by qPCR. The results are presented as the percentage of integrated virus by comparison to the untreated condition.
